# CONTENT VALIDATION OF EDUCATIONAL MATERIAL ON HEALTHY EATING FOR CHILDREN UNDER TWO YEARS OF AGE

**DOI:** 10.1590/1984-0462/;2018;36;2;00007

**Published:** 2018-01-08

**Authors:** Zilda Maria T. Ribeiro, Maria Angélica Spadella

**Affiliations:** a Faculdade de Medicina de Marília, Marília, SP, Brasil.

**Keywords:** Infant nutrition, Child care, Nutrition, public health, Health education, Educational and promotional materials, Nutrição do lactente, Cuidado da criança, Nutrição em saúde pública, Educação em saúde, Materiais educativos e de divulgação

## Abstract

**Objective:**

To validate the content of an educational material aimed at mothers and caregivers on healthy feeding for children less than two years of age.

**Methods:**

Quantitative study for content validation of an educational material containing three educational modules and respective folders, elaborated on the basis of official references for healthy feeding for children under two years old and adapted to the Brazilian context. Content validation was made through consensus conference in two stages by seven experts.

**Results:**

In the first stage, an individual and anonymous evaluation was made and the items analyzed by the seven experts averaged seven or more, with standard deviation below three. However, some items in the educational modules (operational and adherence) and Leaflet I (motivation and culture) reached cut-off values. The second stage involved a face-to-face meeting with five of the seven experts, expressing their opinions and seeking for a new consensus. The items whose results were close to the cut-off had an expressive increase in importance and consensus level after the meeting.

**Conclusions:**

The quantitative data obtained after the consensus meetings were superior to the predefined cut-offs, and the content of the proposed educational material was consensually validated by all participating experts. The consensus conference was an efficient methodological technique to build and validate educational instruments.

## RESUMO

**Objetivo:**

Validar conteúdo de um material educativo sobre alimentação saudável para crianças menores de dois anos orientado às mães e aos cuidadores.

**Métodos:**

Estudo quantitativo que envolveu a validação de conteúdo do material educativo, constituído por três módulos educacionais e seus respectivos folhetos, os quais foram elaborados com base em referenciais oficiais sobre alimentação saudável para crianças menores de dois anos adequados ao contexto brasileiro. Para validar o conteúdo concebido, empregou-se a conferência de consenso, em duas etapas, junto a sete especialistas.

**Resultados:**

Na primeira etapa, de avaliação individual e anônima, os itens analisados pelos sete especialistas obtiveram média igual ou superior a sete, com desvio padrão inferior a três. Porém, itens dos módulos educacionais (operacional e adesão) e do Folheto I (motivação e cultura) alcançaram valores limítrofes ao critério de corte adotado. Na segunda etapa, em reunião presencial com cinco dos profissionais participantes, cada especialista pôde expressar suas considerações, visando à busca de novo consenso. Os itens com notas próximas do limite de corte obtiveram, após o debate, aumento expressivo de valor no grau de importância e nível de concordância dos especialistas.

**Conclusões:**

Os dados quantitativos obtidos após as etapas da conferência de consenso foram superiores ao limite de corte, sendo o conteúdo do material educativo proposto validado de forma consensual pelos especialistas participantes. A conferência consensual empregada revelou ser uma técnica metodológica eficiente para a construção e a validação de instrumentos educativos.

## INTRODUCTION

In the last decades, major changes in morbidity and mortality rate patterns have occurred in Brazil due to the epidemiological, demographic and nutritional transition processes.^1^ ^,^ ^2^ Chronic noncommunicable diseases in adults is a chief health problem, accounting for 72% of death causes among Brazilians.^1^ Among other factors, these diseases are related to eating habits, which are acquired in the first years of life and extend into adulthood.^3^

Changes in family organization, lack of time to prepare meals at home and the practicality of industrialized foods have led to important changes in the population feeding standards, with dominance of ultraprocessed food intake to the detriment of *in-natura* and minimally processed products. The peculiarities of these foods, such as excess sodium, sugars and fats, chemical additives and the absence or reduced amounts of dietary fiber can cause deleterious effects on health, such as obesity, diabetes, hypertension, intolerances and allergies included.^4^ It is even worse when these products are offered to children starting from their first months of life, for it leads to overweight and obesity since childhood,^5^ ^,^ ^6^ especially when combined with early weaning and extemporaneous introduction of complementary feeding. Data from the National Health Survey conducted in 2013^7^ and published in 2015, shows 60.8% of children under the age of five eating industrialized cookies, crackers or cakes, and 32.3% consuming soda or artificial juice.

The development of eating habits during childhood are determinant for the pattern of food consumption in adult life. Therefore, intervening from birth to promote healthy eating habits is part of strategies for the prevention of chronic diseases. Thus, it is of fundamental importance to direct actions that support healthy eating for mothers and caregivers of children under two years of age.

The Ministry of Health (MS), in order to promote healthy eating habits in Brazil, has been working on policies and programs that guide healthy eating practices since childhood in form of important actions aimed at food and nutrition education, within the scope of the Brazilian Public Health System (SUS). However, making food prescriptions feasible is a challenging task for health teams, since this process involves social, cultural, economic and religious aspects in addition to biological ones, and it is even more evident when aimed at two-year-olds because of the need for wide orientation from parents, relatives, and caregivers, who end up being models of habits and attitudes for the child.^8-^ ^10^

Considering that basic care is the main gateway to the health care network, the proposal of an educational material to be implemented along the actions by health teams, providing stimuli to families as to adequate and healthy food practices, it may impact on the food consumption pattern of children, thus constituting a measure of health promotion and better quality of life.^11^ ^,^ ^12^ ^,^ ^13^

Against this backdrop, this study aimed to validate the content of an educational material about healthy feeding aimed for mothers and caregivers of children aged zero to two years old.

## METHOD

Quantitative study, whose design involved: conception and validation of educational material on healthy food for children under two years with the help of specialists. Conduced in compliance with norms and regulatory guidelines for research involving human beings and approved by the Research Ethics Committee of the Medical School of Marília (Famema), CAAE 48246515.6.0000.5413.

After reviewing the literature on the databases Latin American and Caribbean Literature in Health Sciences (Lilacs), Medline (PubMed), Digital Library of Theses and Dissertations (BDTD), Electronic Electronic Library Online (SciELO) and on webpages of the Brazilian Ministry of Health, the Brazilian Society of Pediatrics and the Pan American Health Organization (PAHO)/World Health Organization (WHO), an educational material consisting of educational modules and leaflets has been developed, with emphasis to official information on healthy diet for children under two years of age and appropriate to the Brazilian context.

The modules were organized to encompass a teaching plan with goals, definition of topics, didactic-pedagogical resources, methodology, execution schedule, duration of activities and process evaluation. The guiding axis of contents was the analysis of dietary guidelines, programs and policies established by the Ministry of Health, the Brazilian Society of Pediatrics, WHO, and PAHO.^8^ ^,^ ^9^ ^,^ ^10^ ^,^ ^14^ ^,^ ^15^ ^,^ ^16^

The work strategy suggested in the teaching plan for the development of actions aimed at the target population had two moments: theoretical, dialogic and problematizing approach of themes, based on the theoretical and methodological reference of popular education,^17^ ^,^ ^18^ and practical activities in kitchens adapted for workshops, with participants being invited to compose/prepare recipes suitable for children according to age group. These activities propose that the facilitator use an active teaching-learning method, the talk-wheel technique and the guidelines of the article “The groups in basic health care”, published by the Ministry of Health in the booklet collection “HumanizaSUS”.^19^

Considering the relevance of group actions as strong health practices, the work option for modules’ activities, including mothers and/or caregivers, was the closed group, which determines a pre-established number of participants and meetings, programming and objectives, bringing health promotion and education, and also disease prevention activities into its scope.^19^

The teaching plan of each module also suggests that the activities be evaluated formatively, in process, through participants’ statements during and/or at the end of the meetings. These would be the moments where they would be encouraged to point out positive and negative points, suggest changes, self-assess, evaluate peers and facilitators, talk about the repercussions of group actions on household eating practices, indicate whether there is a change taking place as to eating habits after incorporating new knowledge, and suggest themes or ways of approaching activities to come. Indirect evaluation of educational actions’ repercussions is also suggested, examining growth curves of children in each participating family that are filled in during childcare appointments.

Based on the literature on infant nutrition and the Ministry of Health guide “Ten Steps to Healthy Eating: Food Guide for Children Under Two: A Guide for Health Care Professionals in Basic Care” (freely translated into English), published in 2013^9^, leaflets relating to modules’ themes and aimed as support material for the activities were developed. In this production, we considered the premises for building and bringing effectiveness to health educational materials as to content, language, illustration, organization, layout, learning and motivation.^20^ ^,^ ^21^ ^,^ ^22^ ^,^ ^23^The process of layout creation, formatting, and illustration of the leaflets was carried out by a professional graphic artist.

The content of the educational material was validated at a consensus conference,^24^ a mixed technique that aims to reach a decision in common agreement between two or more parties, conciliating open discussion and anonymity of specialists in a viable logistics.^24^ ^,^ ^25^ ^,^ ^26^ Each module and respective leaflets involved two stages-distance and face-to-face-in a meeting with professionals for open discussion of issues presented.

The selection of experts’ board was based on a convenience sample composed of seven professionals: two pediatricians, one nutrologist nurse, one homeopathic pediatrician, one pediatric nurse specializing in pediatrics and childcare, one PhD pediatrician, one MD in nutrition, and one postdoctoral fellow. Six of the specialists were also professors. Inclusion criteria was: clinical experience in the fields of nutrition and/or childcare, experience in monitoring growth and development of children under two years of age while guiding mothers ­and/­or caregivers. The average professional experience of participants was 25 years.

The first stage of the consensus conference aimed at agreement of opinions between specialists, preserving the anonymity of answers. Preliminary versions of the educational modules and leaflets were handed or sent over (online and postal mail) to specialists along with an informed consent form, with indication of a 30-day period for return. Individual evaluation of materials and completion of the specialist characterization form were requested. The evaluation tool for the educational modules, adapted from Sobral and Santos,^27^ encompassed the following: conceptual, didactic-pedagogic, operational and adherence features. The experts analyzed the evaluation/dimension items, assigning grades from zero to ten, in which zero corresponded to complete disagreement and ten to total agreement. In order to evaluate the leaflets, the experts filled in another instrument of evaluation, adapted from Sousa and Turrini,^28^ composed of the analysis levels: content, language, illustrations, layout, motivation, and culture. Scores from zero to ten were also assigned to each item. Both instruments had three open questions for suggestion of inclusions, revisions and/or exclusions, indication of errors and/or misunderstandings.

The evaluations by specialists were analyzed and the scores were compiled into an Excel spreadsheet (Microsoft, Corporation, USA), with calculation of means of each item in order to verify the importance/appropriateness attributed, as well as standard deviations to estimate the degree of consensus between the specialists.^26^ As per Souza et al.,^24^ cut-off values were: mean ≥7 as an important/adequate item; mean <7 as minor/appropriate item; standard deviation <3 as consensual item; and standard deviation ≥3 as nonconsensual item. The data matrix then guided the improvement process of the preliminary versions.

The second stage of the consensus conference was a face-to-face meeting with all experts, where their consent was requested for the recording of the debate. First, the consolidation of evaluations obtained in the first stage was presented with the grades assigned grades, in addition to the revision suggestions. Afterwards, the debate was opened and each of them could express their understanding about the materials proposed, their arguments in support or against it, as well as to make suggestions, always aiming to deepen the discussions and seek consensus. The debate was organized according to each dimension/level of evaluation, starting with the modules and then the leaflets. After the discussion, the specialists were again asked to complete the assessment and attribute grades to items. Based on the second data matrix, the reformulation suggestions were sent for final material enhancement.

The consensus conference was conducted as recommended by Souza et al.^24^, with the development of three stages to reach consensus in a proposal, two at distance (to preserve anonymity) and one in open discussion of presented matters between specialists. However, during the second stage, the experts considered the implementation of a third anonymous evaluation unnecessary, since they unanimously felt that a new round of evaluations was not necessary given the high quality of the materials.

## RESULTS

**Chart 1:**
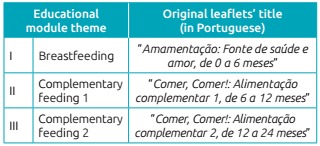
Content defined for each module and correspondent leaflet.

**Figure 1:**
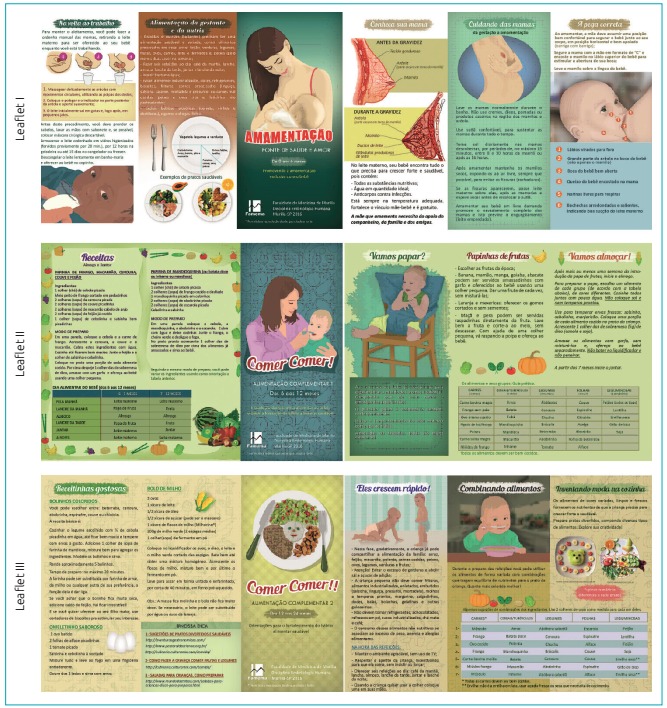
Leaflets for each educational module.

The material’s title is “Promoting a healthy eating habit formation in children under two years” (freely translated into English) and is made up of three educational modules and respective supporting leaflets, including content guiding child feeding process specific to age groups of 0 up to 24 months, in addition to the approach for pregnant women in the last trimester (Chart 1, Figure 1).

The first step of the consensus conference was scheduled within 30 days and had the participation of seven invited experts. There was no need to resubmit the preliminary versions of materials. All evaluation tools were returned with analyses duly completed and open questions accordingly filled in.

The data matrix obtained at this stage showed significant means and standard deviations for each item evaluated/domains of both educational modules and leaflets, with an average of seven or more. In addition, all items were sought to be agreed between specialists, with standard deviations less than three (Tables 1 and 2). Despite this, some items had averages close to seven and standard deviations equal to or greater than two, thus bordering the cut-off criteria. In educational modules, these items were related to operational and adherence domains regarding schedule and workload of activities proposed or frequency to participate and motivation of target public. Only Leaflet I had items with standard deviation values close to cut-off for levels of motivation and culture, in which the content was assessed to solve doubts by the target population according to knowledge/culture levels.

**Table 1:** Mean and standard deviation for items assessed by experts in each educational module, according to validation steps.

Items assessed	Educational module I				Educational module II				Educational module III			
	1^**st**^ step		2^**nd**^ step		1^**st**^ step		2^**nd**^ step		1^**st**^ step		2^**nd**^ step	
	M	SD	M	SD	M	SD	M	SD	M	SD	M	SD
1. Concept domain												
1.1 The themes and content proposed are relevant for the promotion of healthy eating aimed at children aging less than two	9.6	0.8	9.6	0.8	9.8	0.3	9.8	0.4	9.5	0.5	9.8	0.4
1.2 The themes and content proposed are adequate to the target audience (mothers and caregivers of children aging less than two)	9.3	1.0	9.8	0.4	9.2	0.9	9.8	0.4	9.2	0.7	9.8	0.4
1.3 The themes and content proposed are enough to supply the target audience’s needs	8.8	1.3	9.8	0.4	9.0	1.1	9.8	0.4	9.0	1.1	9.8	0.4
1.4 The themes and content proposed allow cognitive ownership by the target audience about healthy feeding for children under the age of two	8.6	1.0	9.4	0.8	9.1	0.6	9.8	0.4	9.0	0.8	9.8	0.4
1.5 The depth of themes proposed is suitable for the target audience	9.3	1.0	9.4	0.8	9.1	1.0	9.8	0.4	9.0	1.1	9.8	0.4
2. Didactic/pedagogical domain												
2.1 The program content of each module is clear and objective	9.3	1.0	9.8	0.4	9.4	0.5	9.8	0.4	9.1	0.6	9.8	0.4
2.2 There is coherence between modules’ objectives and program content	9.1	0.9	9.8	0.4	8.7	0.9	9.8	0.4	8.8	0.6	9.8	0.4
2.3 The teaching strategies are suitable for the target audience	8.8	1.3	9.8	0.4	9.1	1.0	9.8	0.	9.1	1.0	9.8	0.4
2.4 The learning activities proposed allow autonomous learning	8.0	1.2	9.8	0.4	8.8	0.6	9.8	0.4	8.7	0.7	9.8	0.4
2.5 The support material proposed for use during activities favors understanding of the modules’ content	8.6	1.0	9.6	0.5	8.8	1.0	9.8	0.4	8.7	1.2	9.8	0.4
2.6 Didactic resources are easily understandable and foster learning by the target audience	8.8	1.3	9.6	0.5	8.7	1.1	9.8	0.4	8.8	1.0	9.8	0.4
2.7 References used are pertinent and representative	9.3	1.0	9.8	0.4	8.8	1.0	9.8	0.4	9.0	1.1	9.8	0.4
2.8 Individual and collective evaluation process is adequate	8.3	1.0	9.6	0.5	8.8	1.0	9.8	0.4	8.8	1.0	9.8	0.4
3. Operational domain												
3.1 The execution timetable of modules is adequate	8.5	2.3	9.6	0.5	8.4	2.6	9.4	0.8	8.5	2.5	9.6	0.5
3.2 The hourly load of modules is compatible with activities proposed	7.6	2.8	9.6	0.5	7.7	2.5	9.8	0.4	7.7	2.2	9.8	0.4
3.3 The place where activities of each module are conducted is adequate	8.8	1.6	9.8	0.4	7.8	2.5	9.8	0.4	8.4	1.2	9.8	0.4
4. Adhesion domain												
4.1 The educational strategy proposed will encourage and motivate the participation of target audience	7.6	1.6	9.2	0.8	8.2	1.6	9.6	0.5	7.8	1.4	9.6	0.5
4.2 Activities proposed in modules allow frequent activity practice by the target audience	7.0	2.0	9.0	0.7	8.2	1.6	9.2	0.8	7.8	1.4	9.4	0.5

M: mean; SD: standard deviation; M≥7: item considered important/adequate; SD<3: consensual item.

**Table 2:** Mean and standard deviation for items assessed by experts in each leaflet, according to validation steps.

Items assessed	Leaflet I				Leaflet II				Leaflet III			
	1^**st**^ step		2^**nd**^ step		1^**st**^ step		2^**nd**^ step		1^**st**^ step		2^**nd**^ step	
	M	SD	M	SD	M	SD	M	SD	M	SD	M	SD
1. Content												
1.1 The content covered is relevant for the promotion of healthy eating aimed at children aging less than two	9.8	0.3	9.6	0.5	9.8	0.3	9.4	0.5	9.5	0.5	9.6	0.5
1.2 The content is suitable for the target audience (mothers and caregivers of children aging less than two)	9.0	1.5	9.8	0.4	9.4	0.7	9.6	0.5	9.4	0.7	9.6	0.5
1.3 The content is enough to supply the target audience’s needs	8.4	2.2	9.6	0.5	8.7	1.2	9.6	0.5	8.8	1.4	9.6	0.5
1.4 The content can be easily applied in the target audience’s daily routine	9.3	1.2	9.8	0.4	9.1	0.6	9.6	0.5	9.2	0.7	9.6	0.5
2. Language												
2.1 Writing style is compatible with the target audience	8.5	0.9	9.8	0.4	9.2	0.7	9.8	0.4	9.4	0.5	9.6	0.5
2.2 Writing style is attractive	9.0	1.1	9.8	0.4	9.2	0.7	9.8	0.4	9.2	0.7	9.6	0.5
2.3 The language used is clear and objective	8.8	1.0	9.8	0.4	9.4	0.5	9.8	0.4	9.4	0.5	9.6	0.5
3. Illustrations												
3.1 Illustrations are adequate to and match the theme of the support material	9.1	1.2	9.8	0.4	9.1	1.0	9.8	0.4	9.5	0.7	9.8	0.4
3.2 Illustrations are clear and allow easy understanding	9.1	1.4	9.8	0.4	9.4	0.7	9.8	0.4	9.5	0.7	9.8	0.4
3.3 The number of illustrations is content-suitable in support materials	9.8	0.3	9.6	0.5	9.4	0.5	9.8	0.4	9.5	0.7	9.8	0.4
4. Layout												
4.1 The font type eases reading	9.5	0.5	9.8	0.4	9.2	0.4	9.8	0.4	9.2	0.7	9.8	0.4
4.2 Colors are adequate and ease reading	9.4	0.7	9.8	0.4	9.2	0.4	9.8	0.4	9.1	1.0	9.8	0.4
4.3 Visual composition is attractive and organized	9.5	0.7	9.8	0.4	9.2	0.4	9.8	0.4	9.5	0.5	9.8	0.4
4.4 The size (dimensions) and number of pages of the support material are appropriate	9.4	0.7	9.8	0.4	9.4	0.5	9.8	0.4	9.4	0.5	9.8	0.4
4.5 Copy layout is adequate	9.7	0.4	9.8	0.4	9.4	0.5	9.8	0.4	9.5	0.5	9.8	0.4
4.6 Font size in headings and copy are adequate	9.4	0.5	9.8	0.4	9.4	0.5	9.8	0.4	9.5	0.5	9.8	0.4
5. Motivation												
5.1 The content is motivating and encourages full reading	9.2	0.7	9.8	0.4	9.4	0.5	9.8	0.4	9.4	0.5	9.8	0.4
5.2 The content awakens interest in readers	9.1	0.6	9.8	0.4	9.4	0.5	9.8	0.4	9.4	0.5	9.8	0.4
5.3 The content solves doubts, clears things up, and educates the target audience	8.0	2.0	9.4	0.5	8.8	1.0	9.6	0.5	9.1	1.0	9.4	0.5
6. Culture												
6.1 The copy is appropriate to the target audience and the various knowledge-level profiles	8.2	2.0	9.4	0.5	9.0	1.1	9.6	0.5	9.2	1.1	9.4	0.5

M: mean; SD: standard deviation; M≥7: item considered important/adequate; SD<3: consensual item.

When analyzing answers to open questions in the evaluation instruments, the grades assigned by specialists were in conformity with these measurements. The suggestions that were in line with the objective of the study were accepted. The whole process contributed to enriching the proposal, especially in domains whose scores were borderline, since evaluations/suggestions came from professionals who share the interest of qualifying children health care.

The second step of the consensus conference took place with five out of seven experts, ensuring representativeness of all specialties (Pediatrics, Nursing and Nutrition). In an open debate, each expert expressed their considerations aiming to deepen the discussions in search of a new consensus. They then proceeded to complete the evaluations. The data in the second matrix indicated that items with scores close to cut-off in the first stage had significantly increased in importance and consensus level between the specialists (Tables 1 and 2). This second evaluation assured a new opportunity for material improvement, with subsequent upgrade coherent with and adapted to the predefined purposes.

## DISCUSSION

The choice of approach to this material about healthy eating habits has emerged from the concern about the current nutritional status of children, where overweight and obesity prevail and which differs a lot from few decades ago, when malnutrition consumed children’s health. The current situation observed in clinical practice is intriguing, with frequent feeding errors coexisting with nation-wide public policies on healthy eating so well-delineated and disclosed in manuals.

In this context, our material was prepared in the light of scientific evidence on infant feeding process management, from breastfeeding to complementary feeding, focusing on the ages from 0 to 2 years, in order to make it stand out as an instructive material that is also feasible for mothers and caregivers, aiming at the formation of healthy eating habits.^8-^ ^10^ ^,^ ^14^

Content validation of the educational material was reached in a consensus by a board of experts highly qualified in childcare and child nutrition, with technical background in clinical practice and acting in the monitoring of child growth and development. A rich, effective, objective compilation of the food policies by the Ministry of Health was developed,^8^ ^,^ ^9^ ^,^ ^10^added with the offer of simple and decoded instructions on how to make dietary orientation for caregivers of infants, walking basic health care professionals through steps for its applicability.

Mean and standard deviation values obtained in the first stage of the consensus conference showed, for the most part, the appropriateness of the proposed educational material, so the procedure was reinforced in the stage of face-to-face meetings. However, the results of the first stage were central to the reformulation of the modules regarding operational and adhesion, with borderline averages. The experts judged that the three-hour workload to cover modules was too extensive and tiring, and this could also interfere with the motivation of the target population. Therefore, the reformulated version of the material proposed a reduction in hours for each module, which was considered appropriate by specialists in common agreement, in the second stage of the conference.

Regarding leaflets, the close-to-cut-off standard deviation values obtained in the first stage of the conference for Leaflet I guided the modifications of the preliminary version as to motivation and culture. In this way, certain terms were replaced in order to make language simpler and easier to understand across different levels of education and culture. The new version of was then considered appropriate by the experts at the open discussion meeting.

In addition, the broad analysis by experts at the consensus conference guided the refinement of the proposed educational material, making it clear that their power in actions to promote the early formation of a healthy eating habit depends on how the guidelines are passed on to the target population. For this reason, the proposed work with the suggested population is the active, dialogic, and activity-centered method based on Paulo Freire’s popular education referential.^17^ ^,^ ^18^ This proposal of work is aligned with the purposes of basic care: reaching portions of the population that need collective educational and transformative actions, involving change of habits, in the case of feeding, while focusing on learning. In this direction, the role of the facilitator should be well delineated and could be played by anyone in the health care team - even by nursery and preschool professionals or child caregivers at their households -, since the basic requirement is simply to be willing to do so.^29^ ^,^ ^30^

The two-stage consensual conference^24^ was an important tool for validating the content of the proposed material on healthy eating for children under two years. Through the scientific methodology employed, the build and validation of content conceived by experts of the area ensured its suitability to the target public’s actual needs. It is therefore expected that this study can encourage and support other researchers to elaborate and corroborate instruments aimed at health education, in a way they are developed with scientific rigor and applied to the population to supply their needs, not depending only on the scope defined by who creates them.

Although this study has depicted the careful process of content validation for a proposition of educational material, not being applied to the target audience is a limitation. This prevents us from evaluating the full understanding and effectiveness of the information addressed in modules and leaflets in practice. Therefore, it is expected that the next steps will be towards the applicability of the material to the target population, especially in the health education actions of the primary care teams, since it may contribute with qualification of care while encouraging healthy eating practices to promote health, improve quality of life and prevent diseases.
